# Placental Adhesion Subsequent to Uterine Preservation via Resuscitative Endovascular Balloon Occlusion of the Aorta: A Case Report

**DOI:** 10.7759/cureus.75732

**Published:** 2024-12-15

**Authors:** Koshiro Nagata, Toyofumi Hirakawa, Daisuke Izuchi, Daichi Urushiyama, Fusanori Yotsumoto

**Affiliations:** 1 Department of Obstetrics and Gynecology Faculty of Medicine, Fukuoka University, Fukuoka, JPN

**Keywords:** fertility preservation, hemorrhagic shock, placenta accrete, primipara, resuscitative endovascular balloon occlusion of the aorta

## Abstract

An adherent placenta is a life-threatening condition that impairs the mother’s life owing to hemorrhagic shock and disseminated intravascular coagulation. Profound hemorrhage resulting from placental abruption is often managed using hysterectomy to preserve the mother’s life, although the consequent loss of fertility can be devastating, particularly in younger women. Thus, strategies that facilitate fertility preservation while effectively controlling hemorrhage should be considered viable alternatives. Resuscitative endovascular balloon occlusion of the aorta (REBOA) is commonly performed in patients experiencing traumatic hemorrhagic shock; however, its application in obstetric cases remains infrequent. Herein, we report a case in which REBOA was used to control hemorrhage following the manual removal of an adherent placenta, with the aim of preserving the uterus. A 28-year-old woman presented to our hospital with hemorrhagic shock owing to extensive bleeding from an adherent placenta. As the patient was a young, primiparous woman with a strong desire to preserve her uterus, we opted for manual placental removal. However, manual removal poses a risk of exacerbating the hemorrhage. Therefore, REBOA was performed by emergency physicians to reduce bleeding during placental abruption, and intrauterine balloon tamponade was used to achieve hemostasis without compromising the patient's condition. The use of REBOA in the management of placenta accreta not only improves survival rates but may also provide crucial time for fertility-preserving interventions.

## Introduction

Adherent placenta is a pathological condition characterized by the direct infiltration of chorionic villi into the myometrium from a defective site of the abruption membrane within the uterine cavity, persisting unabated postpartum [1]. Vigorous manual detachment of the placenta may precipitate shock and disseminated intravascular coagulation syndrome secondary to profuse hemorrhage, exacerbating maternal prognosis [2-4]. Consequently, hysterectomy is frequently performed as a life-saving intervention for the mother; however, the resulting loss of fertility is both emotionally and physically distressing for the woman. Therefore, procedures that offer the potential for fertility preservation while effectively managing hemorrhage should be considered viable options, although no clearly definitive method currently exists. Resuscitative endovascular balloon occlusion of the aorta (REBOA) denotes a procedure entailing transient occlusion of the aorta to facilitate controlled placental extraction. As a minimally invasive maneuver, REBOA temporarily obstructs the aorta, augmenting cerebral and coronary perfusion proximal to the occlusion, thereby enhancing survival rates through decreased distal pressure and hemorrhage. This transient aortic occlusion aids in normalizing blood pressure and affording adequate time for hemorrhage cessation [5,6]. While conventionally employed in instances of traumatic hemorrhagic shock, its obstetrical application is relatively infrequent. Herein, we delineate a case in which the utilization of REBOA during manual removal of a clinically adherent placenta resulted in hemostasis, ensuring effective hemorrhage control and successful uterine preservation.

## Case presentation

The patient is a 28-year-old primiparous woman with no significant medical history. Following confirmation of a spontaneous pregnancy, she received routine prenatal care from her previous physician, and the pregnancy progressed without complications. The patient went into labor at 40 weeks and 5 days of gestation, delivering a 3,366 g infant via spontaneous vaginal delivery in the cephalic presentation. The placenta was not expelled spontaneously within 60 minutes postpartum, and an attempt at manual removal was made, but it remained undelivered. During this time, active hemorrhage persisted, with a total blood loss of 1,868 g within 60 minutes postpartum. Her blood pressure was 99/72 mmHg, pulse rate was 152 bpm, and her Shock Index was calculated at 1.5. She was urgently transferred to our hospital with a diagnosis of hemorrhagic shock and adherent placenta. The patient arrived at our hospital 111 minutes post-delivery. Upon arrival, her vital signs were consciousness of 15 points (E4V5M6) on the Glasgow Coma Scale, blood pressure of 70/40 mmHg, heart rate of 164 beats per minute (Shock Index 2.3), respiratory rate of 17 breaths per minute, and SpO_2_ of 97% (oxygen 5 L/min). Vaginal speculum examination revealed no active bleeding. Transabdominal ultrasonography showed residual placenta in the uterus and continuous blood flow from the uterine fundus to the placenta, with no evidence of retroplacental clear space loss or placental lacunae. Blood tests showed a hemoglobin level of 8.3 g/dL, platelet count of 297,000 /μL, prothrombin time of 12.7 seconds, activated partial thromboplastin time of 25.1 seconds, fibrinogen level of 316 mg/mL, and D-dimer level of 16.5 μg/mL (Table 1).

**Table 1:** Laboratory results

Investigation result	Value	Normal range
White blood cell count	27.7 × 10^3^/μL	3.3–8.6 × 10^3^/μL
Hemoglobin	8.3 g/dL	11.6–14.8 g/dL
Platelet count	297 × 10^3^/μL	158–348 × 10^3^/μL
Hematocrit level	26.3%	35.1–44.4%
Mean cell volume	97.4 fL	83.6–98.2 fL
Mean cell hemoglobin level	30.7 pg	27.5–33.2 pg
Mean cell hemoglobin concentration	31.6 g/dL	31.7–35.3 g/dL
Neutrophil ratio	92.0%	50–70%
Eosinophil ratio	0.0%	1–5%
Basophil ratio	0.1%	0–1%
Lymphocyte ratio	3.2%	20–40%
Monocyte ratio	4.7%	1–6%
Prothrombin time	12.7 s	9.8–12.1 s
International normalized ratio	1.13	0.8–1.1
Activated partial thromboplastin time	25.1 s	24.0–34.0 s
Fibrinogen	316 mg/dL	200–400 mg/dL
Antithrombin Ⅲ	67%	80–130%
D-dimer	16.5 μg/mL	≤1.0 μg/mL
Albumin level	2.1 g/dL	4.1–5.1 g/dL
C-reactive protein	0.95 mg/dL	0–0.14 mg/dL
Urea nitrogen	8 mg/dL	8–20 mg/dL
Creatinine	0.71 mg/dL	0.46–0.79 mg/dL
Sodium level	139 mmol/L	138–145 mmol/L
Potassium level	3.7 mmol/L	3.6–4.8 mmol/L
Chloride level	110 mmol/L	101–108 mmol/L
Calcium level	7.8 mg/dL	8.8–10.1 mg/dL
Total bilirubin	0.5 mg/dL	0.4–1.5 mg/dL
Aspartate aminotransferase	23 U/L	13–30 U/L
Alanine aminotransferase	6 U/L	7–23 U/L
Lactate dehydrogenase	218 U/L	124–222 U/L
Alkaline phosphatase	150 U/L	38–113 U/L
γ-Glutamyl transpeptidase	6 U/L	9–32 U/L
Cholinesterase	180 U/L	201–421 U/L
Creatine kinase	407 U/L	41–153 U/L
Amylase	57 U/L	44–132 U/L
Glucose	98 U/L	73–109 U/L

**Figure 1:**
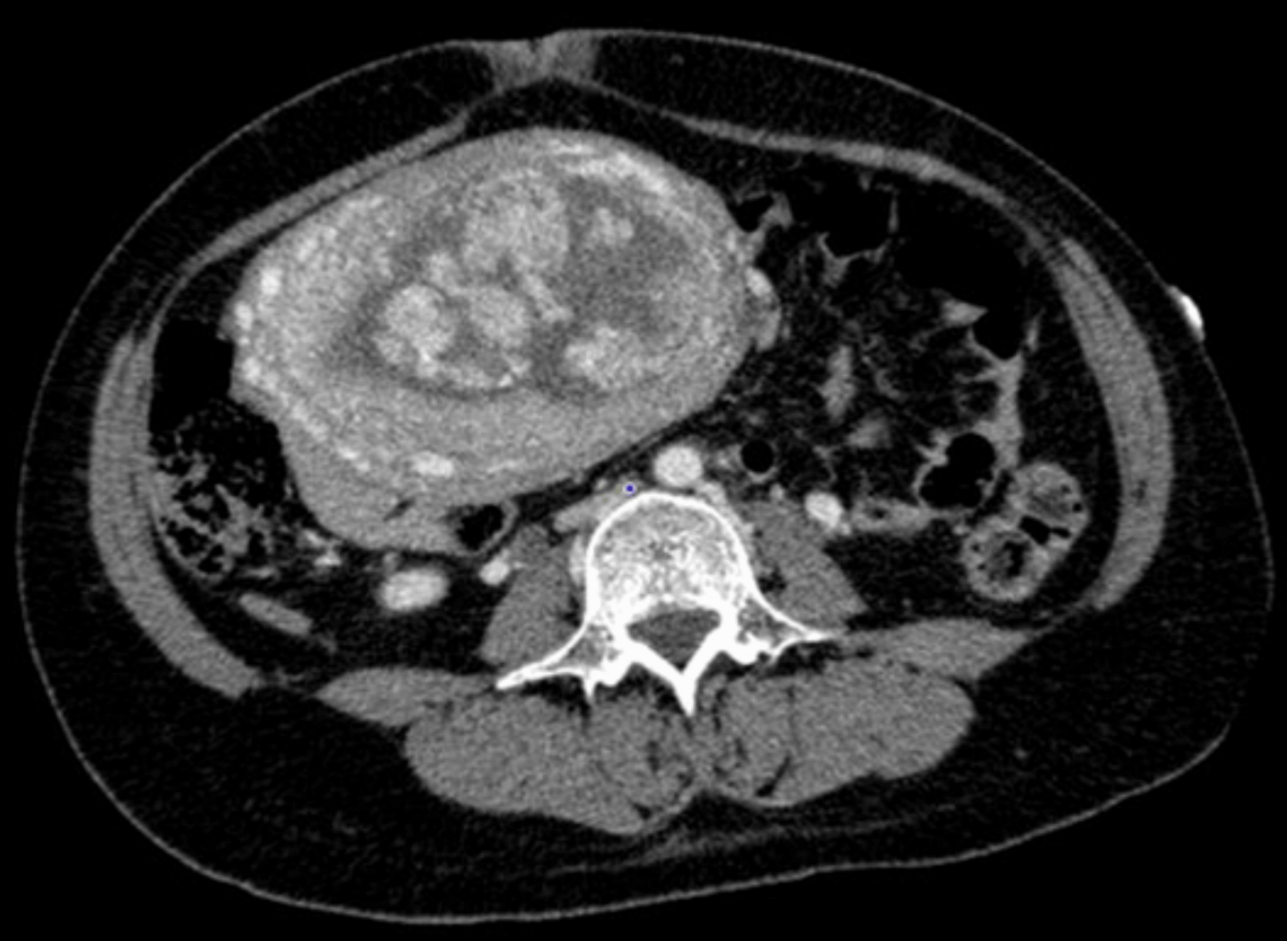
Computed tomography scan (axial section) The placenta is adherent to the uterine fundus. There is no leakage of the contrast medium into the uterine cavity.

Liver and kidney function tests were normal. A contrast-enhanced computed tomography (CT) scan showed no contrast leakage into the uterine lumen (Figure 1).

Hemorrhagic shock was diagnosed, and transfusions of red cell concentrate, fresh frozen plasma, and cryoprecipitate were initiated. Contrast-enhanced CT revealed no leakage of contrast medium into the uterine lumen.

Since the bleeding was well controlled, the patient was a young first-time mother, and there was a strong desire for uterine preservation, we decided to perform a manual removal of the placenta. A REBOA was inserted into the aorta (zone 1) at 27 minutes after arrival at the hospital, and the operation was started under general anesthesia in the Emergency Department of the hospital. The REBOA kept the patient's vital signs stable throughout the procedure. The placenta was partially adhered to the endometrial wall but was delivered without any defect and was considered placenta accreta. Intermittent inflation of the REBOA facilitated the manual removal of the placenta with minimal hemorrhage related to placental abruption, followed by balloon tamponade using a Bakri balloon. Total inflation time for aortic occlusion was 17 minutes, and intraoperative blood loss was 600 mL. Postoperative blood tests revealed a hemoglobin level of 10.7 g/dL, platelet count of 96,000/µL, prothrombin time of 16.2 seconds, activated partial thromboplastin time of 46.8 seconds, fibrinogen level of 151 mg/mL, and D-dimer level of 15.9 μg/mL. Total blood loss from the previous physician was 2,505 g. The total transfusion volume was 14 units of red blood cell concentrate, 16 units of fresh frozen plasma, and 3 g of fibrinogen concentrate (FibrinogenHT) according to the massive transfusion protocol [7].

The Bakri balloon was removed the day after surgery, and there was no bleeding from the uterine lumen. Transvaginal ultrasonography revealed no evidence of placental remnants. Blood tests showed improvement in her anemia and coagulation system (Table 1), and her general condition was good; therefore, she was transferred to her previous doctor on the seventh postoperative day.

## Discussion

Adherent placenta arises from anomalous infiltration of placental tissue into the uterine wall, categorized as accreta, increta, and percreta [1]. The incidence of placenta accreta is estimated at approximately 1 in 2,500 deliveries, predisposing to obstetric crisis hemorrhage and contributing to perinatal mortality [2]. Ordinarily, the placenta detaches effortlessly and is expelled alongside the membranes following fetal delivery. However, in the absence or thinning of the uterine decidua, trophoblastic tissue may directly infiltrate the myometrium, culminating in an adherent placenta, particularly when increta or percreta is forcefully removed, precipitating abnormal hemorrhage at parturition. Notably, hemorrhage stemming from placenta accreta previa dissection is rapid and copious, portending grave maternal outcomes and maternal mortality risks [3]. Consequently, upon diagnosis of an adherent placenta, total hysterectomy without manual removal of the placenta is the most definitive approach to avert aberrant bleeding. Nonetheless, in cases where uterine preservation is strongly desired or hysterectomy proves challenging, spontaneous resorption or delivery without placental detachment may be contemplated. However, this strategy entails infection risk and swift abnormal hemorrhage onset during the waiting period [8]. Moreover, the hemorrhagic propensity of an adherent placenta is formidable, with mean blood loss approximating 3,000-5,000 mL [4]. Consequently, unrelenting bleeding may swiftly prove fatal, compelling patients to opt for total hysterectomy, notwithstanding the consideration of conservative therapies to safeguard their lives. Nonetheless, total hysterectomy poses a risk of fertility loss and significantly impacts subsequent quality of life. Thus, the potential for uterine preservation arises provided hemostasis can be achieved to secure time against persistent bleeding.

REBOA represents a minimally invasive methodology aimed at enhancing survival rates by deploying a balloon within the aorta, thereby transiently occluding it to augment cerebral blood flow and coronary perfusion proximal to the occlusion, while mitigating distal pressure and hemorrhage. This technique serves as an interim measure to normalize blood pressure and afford ample time for hemorrhage cessation; it is not deemed a permanent remedy but rather serves as a bridge to stabilize the patient until definitive hemostatic interventions such as vascular embolization or surgery can be pursued. Primarily utilized in cases of traumatic hemorrhagic shock [5], sporadic reports have emerged in recent years regarding its efficacy in managing bleeding in non-traumatic hemorrhagic shock scenarios, including gastrointestinal bleeding [6]. The placement of the occlusive balloon is contingent upon segmenting the aorta into three zones based on the bleeding source. Zone 1 encompasses the left subclavian aorta, extending from the left subclavian artery to the celiac artery, zone 2 spans from the celiac artery to the renal artery, and zone 3 encompasses the region from the renal artery to the aortic bifurcation. REBOA achieves complete or partial cessation of peripheral blood flow above the occlusion site, thereby curtailing hemorrhage but also inducing ischemia in normal tissue. Given the risk of injury upon reperfusion, use of REBOA should be judiciously limited when complications outweigh therapeutic benefits [9,10]. Prolonged balloon inflation elevates the risk of peripheral organ damage and mortality [11], necessitating strict adherence to an occlusion timeframe of approximately 30 minutes or less. Challenges during REBOA implementation encompass malpositioning and vascular injury, while balloon deflation may trigger the release of inflammatory mediators, embolization, and rebleeding from the injury site. The impact of these adverse events hinges upon institutional technique and management, with adept proficiency in REBOA technique mitigating risks to an acceptable level vis-à-vis its benefits [12]. Nonetheless, opportunities for obstetricians and gynecologists to undertake REBOA insertion are exceedingly scarce, underscoring the imperative for collaborative efforts with emergency physicians for insertion and management of REBOA in clinical practice.

The indications for REBOA have been broadening in recent years, with anticipated use in obstetrics and gynecology. In this field, reports have surfaced regarding its application in obstetric crisis hemorrhage, prophylactic insertion during elective cesarean section for cases of placenta accreta to mitigate intraoperative blood loss, and postoperative hemorrhagic shock in gynecological ailments [4,13]. Particularly noteworthy are the burgeoning reports on REBOA's utility in managing obstetric hemorrhage, a leading cause of perinatal mortality, where its implementation aids in blood loss control. Prophylactic REBOA insertion has been explored in high-risk patients predisposed to massive hemorrhage, notably in cases of adherent placenta, demonstrating significantly diminished blood loss and transfusion rates compared to non-utilizing cohorts [6,14]. Zone 3 REBOA is typically preferred for managing uterine bleeding. A systematic review focusing on the prophylactic use of zone 3 REBOA for placenta previa-complicated elective cesarean section revealed its efficacy in reducing blood loss and transfusions, devoid of REBOA-related adverse events, potentially ameliorating prognosis in patients at risk of fatal postpartum hemorrhage [15]. Moreover, there are reports indicating REBOA's potential in uterine preservation for obstetric crisis hemorrhage [13], with rapid insertion possibly contributing not only to maternal survival but also to fertility preservation.

In the present case, REBOA was employed for adherent placenta and obstetric hemorrhage management, effectively stemming bleeding during and after placental abruption while conserving the uterus. However, the timing of REBOA insertion bears a direct correlation with survival, with delayed insertion linked to decreased survival rates. Notably, the median time for successful REBOA insertion in survivors was reported as 25 minutes compared to 47 minutes in non-survivors [16]. Hence, expeditious REBOA insertion is paramount for treatment efficacy, underscoring the necessity for collaborative intervention with well-trained emergency physicians. Emergency physicians' active involvement in managing obstetric hemorrhage equips them to provide timely REBOA options, potentially serving as a pivotal strategy for global maternal mortality reduction. In this instance, the paramedic performed REBOA insertion was completed within a swift 27 minutes from delivery, indicating rapid intervention. Furthermore, in cases where manual removal of the placenta proves challenging or subsequent measures such as intrauterine balloon tamponade or uterine artery embolization fail to control bleeding, manual dissection with REBOA may emerge as a viable option, especially when uterine preservation is paramount and prompt total hysterectomy is feasible.

## Conclusions

Originally employed for the management of traumatic hemorrhagic shock, REBOA has recently been applied to diverse scenarios encompassing obstetric emergencies such as placenta accreta. Adherent placenta accreta represents a prevalent etiology of obstetric crisis hemorrhage and maternal mortality, where REBOA not only augments life-saving efficacy but also mitigates the necessity for unwarranted hysterectomies. While serving as a transitional measure for definitive therapy, the potential adverse events associated with REBOA should be acknowledged. Nevertheless, through collaborative efforts with emergency physicians and comprehensive knowledge and experience, attendant risks can be mitigated, thereby amplifying the utility of REBOA.
